# Synergy between upregulated small GTPase immunity-associated proteins and lysosomal ATP6V1D in restricting intracellular *Toxoplasma* growth

**DOI:** 10.1128/spectrum.03947-25

**Published:** 2026-04-13

**Authors:** Md Mukthar Mia, Shahbaz M. Khan, Abdur Rehman Azam, Jordan Reilly, William H. Witola

**Affiliations:** 1 Department of Pathobiology, College of Veterinary Medicine, University of Illinois Urbana-Champaign 14589, Urbana, Illinois, USA; 2 Johns Hopkins Bloomberg School of Public Health 25802 , Baltimore, Maryland, USA; Clemson University, Clemson, South Carolina, USA

**Keywords:** *Toxoplasma *inhibition, GIMAP, ATP6V1D, lysosome, Lewis rat

## Abstract

*Toxoplasma gondii* is an obligate intracellular zoonotic protozoan parasite that can cause severe neonatal disease in congenitally acquired infections and severe opportunistic illnesses in immunocompromised individuals. Intracellularly, *T. gondii* evades host cell defense mechanisms by residing within a specialized non-fusogenic membranous compartment known as the parasitophorous vacuole (PV). Notwithstanding this, we have previously shown that upregulation of small GTPase immunity-associated proteins (GIMAPs) during *T. gondii* infection promotes fusion of lysosomes with the PV membrane (PVM), resulting in restriction of parasite replication in the *T. gondii*-refractory Lewis (LEW) rat. Herein, we found that, in addition to GIMAPs, the LEW rat upregulates ATP6V1D, a subunit of the lysosomal vacuolar-type H+-ATPase (v-ATPase) proton pump, in response to *T. gondii* infection. Disruption of ATP6V1D by CRISPR/Cas9 in a rat macrophage (NR8383) cell line impaired v-ATPase function, leading to defective lysosomal acidification and a concomitant increase in intracellular *T. gondii* growth. Intriguingly, overexpression of GIMAP 5 or 6 transgene in the ∆*ATP6V1D* mutant cell line reduced parasite growth by 2.5- and 5-fold, respectively. Comparatively, overexpression of GIMAP 5 or 6 transgene in the wild-type NR8383 cell line had at least threefold more robust effect of reducing parasite growth than in the ∆*ATP6V1D* mutant cell. Together, our findings imply that while GIMAP-induced activation of lysosome translocation to the PVM is important for restricting parasite growth, concomitant upregulation of the ATP6V1D plays an additively significant role through enhanced acidification of lysosomes to activate hydrolytic enzymes that degrade the parasites following PVM fusion.

**IMPORTANCE:**

*Toxoplasma gondii* is a highly prevalent parasite that causes severe illnesses in congenitally infected neonates and in immunocompromised individuals. No completely effective drugs nor vaccines exist against *T. gondii*. Thus, understanding innate mechanisms that resistant hosts employ to orchestrate defenses against the parasite could unveil new strategies for control of toxoplasmosis. Herein, we analyzed transcriptomic data of the *T. gondii*-resistant rat (Lewis) and identified small GTPase immunity-associated proteins (GIMAPs), and lysosome-associated proteins (including ATP6V1D) that are upregulated in response to infection. We investigated the interplay between those proteins and found that upregulated expression of GIMAPs drives lysosomes to fuse with the parasite vacuole, while upregulated ATP6V1D facilitates lysosomal acidification, which is important for activation of degradative lysosomal enzymes. Those events culminated in the restriction of intracellular *T. gondii* growth. Collectively, our findings indicate that upregulation of GIMAPs and ATP6V1D orchestrates synergistic mechanisms that contribute to inhibition of intracellular *T. gondii* growth.

## INTRODUCTION

*Toxoplasma gondii* is a globally prevalent obligate intracellular protozoan parasite capable of infecting nearly all warm-blooded animals, with an estimated 2 billion people chronically infected (1). *T. gondii* is a major cause of reportable foodborne illnesses and is associated with severe neonatal disease in congenitally acquired infections, as well as opportunistic disease in immunocompromised individuals globally (2). Infection can also lead to ocular disease that can manifest as retinochoroiditis or uveitis (3).

*T. gondii* evades host intracellular defense mechanisms by residing within a specialized non-fusogenic membranous compartment known as the parasitophorous vacuole (PV) (4). Host resistance to *T. gondii* relies on a complex interplay of cellular defense mechanisms in which immunity-related GTPases (IRGs), guanylate-binding proteins (GBPs), and the more recently characterized small GTPase immunity-associated proteins (GIMAPs) play pivotal roles (5, 6). While IRGs and GBPs are well-characterized mediators of interferon-gamma (IFN-γ)-induced parasite restriction mechanisms in mice and humans, respectively, GIMAPs have emerged as important modulators of host cell-autonomous immunity, particularly in monocytic cells and macrophages (5, 6).

We have previously shown that, unlike in the Brown Norway rat (*T. gondii*-permissive rat strain), *T. gondii* infection in the Lewis rat (*T. gondii*-refractory rat strain) upregulates the expression of GIMAP 4, GIMAP 5, and GIMAP 6 that translocate to the PV membrane (PVM) (5). Intriguingly, ectopic overexpression of those GIMAPs in a macrophage cell line (derived from the *T. gondii*-permissible Sprague Dawley rat strain) induced localization of lysosomes on the otherwise non-fusogenic PVM, with concomitant restriction of intracellular *T. gondii* proliferation (5). This phenomenon appears to be similar to the role of IRGs that induce lysosomal fusion to the PVM, resulting in the destruction of intra-vacuole *T. gondii* tachyzoites in mouse cells (7–9). Beyond *T. gondii*, GIMAP 5 has been implicated in the induction of lysosomal degradation of other intracellular pathogens, such as respiratory syncytial virus (RSV), through its interaction with the mannose-6-phosphate receptor (M6PR) (10). Furthermore, GIMAP 5 and GIMAP 6 have been shown to restrict intracellular herpes simplex virus 1 replication, with concomitant reduction in viral particle production (11).

The phagosome-lysosome fusion process serves as a mechanism for the destruction of invading intracellular microbes by the lysosomal hydrolytic enzymes that are discharged into the phagosome following fusion (12). Hydrolytic enzymes within lysosomes are activated by the acidic intraluminal environment that is achieved through the action of the vacuolar-type H+-ATPase (v-ATPase) proton pump that drives H+ from the cell cytosol into the lysosome lumen (13, 14). The v-ATPase is a multimeric protein complex present in all eukaryotic cells. It is composed of the V1 (cytosolic) and the V0 (membrane-bound) subcomplexes. The V1 subcomplex includes eight subunits, A–H, with three copies each of the catalytic A and B subunits, three copies of the stabilizing subunits E and G, and one copy each of the regulatory C and H subunits. The V1 subcomplex also includes the D and F subunits that form a central rotor axle that is driven by the hydrolysis of ATP by the catalytic V1A/V1B subunits and acts to power the V0 subcomplex to move protons across the lysosomal membrane into the lumen (13, 14). v-ATPase subcomplex V1 subunit D (ATP6V1D) is required for lysosomal acidification (15, 16). Herein, we investigated the interplay between upregulated GIMAP and ATP6V1D in modulating intracellular *T. gondii* growth and proliferation.

## RESULTS

### Lysosomal pathway-associated genes are preferentially upregulated in Lewis rat following *T. gondii* infection

**Fig 1:**
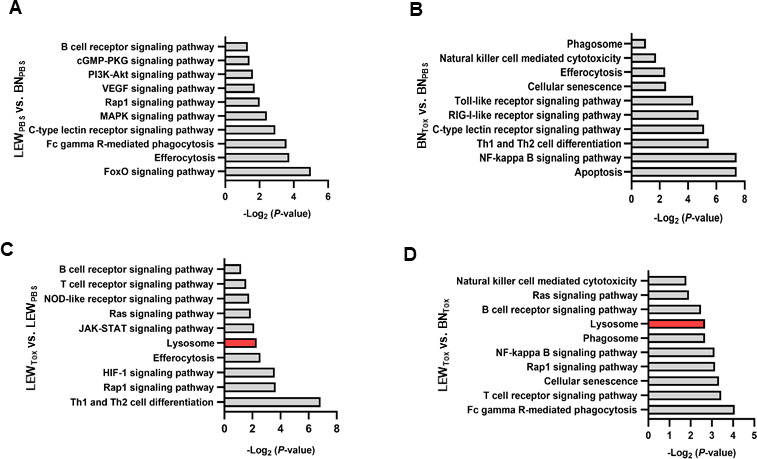
Comparison of the top 10 enriched immune-related pathways in Lewis (LEW) and Brown Norway (BN) rats, with or without *T. gondii* infection. The bulk RNA sequencing data (GEO ID: GSE100203) was analyzed across four different treatments: (**A**) LEW_PBS_ vs BN_PBS_, (**B**) BN_Toxo_ vs BN_PBS_, (**C**) LEW_Toxo_vs LEW_PBS_, and (**D**) LEW_Toxo_ vs BN_Toxo_. Differentially expressed genes (log_2_ FC > 1.20 and adjusted *P*-value < 0.05) were submitted to the DAVID database for KEGG pathway enrichment analysis. The top 10 enriched immune-related pathways were selected based on immunology-associated terms. (**A–D**) Bar graphs delineate the top 10 significantly enriched immune-related KEGG pathways. Red bars indicate enriched genes associated with the lysosomal pathway. BN_Toxo_ and LEW_Toxo_ (*T. gondii*-infected BN and LEW rats, respectively); BN_PBS_ and LEW_PBS_ (uninfected BN and LEW rats, respectively).

We have previously generated bulk RNA sequencing data (GEO ID: GSE100203) by comparing transcriptomes of Lewis (LEW) and Brown Norway (BN) rats, with or without *T. gondii* infection (5, 17). In the present study, we performed an analysis of the RNA sequence data in the GEO repository to determine enriched immune-related pathways in LEW vs BN rats in response to *T. gondii* infection. We observed that the LEW and BN rats differ in their baseline (without *T. gondii* infection) transcriptomes, with the uninfected LEW rat having inherently upregulated immune-associated pathways than the BN rat (Fig. 1A). In response to *T. gondii* infection, compared with their baseline transcriptomes, both the BN and LEW rats depicted distinct enriched immune-associated pathways (Fig. 1B and C). Furthermore, when LEW and BN infected rats were compared, the LEW rat had an upper hand for enriched immune-associated pathways than the BN rat (Fig. 1D). The notable top 10 immune-associated pathways that the LEW rat upregulated in response to *T. gondii*, whether or not in comparison to the infected BN rat, included Rap1 signaling, Ras signaling, T-cell receptor signaling, lysosome, and B cell receptor signaling pathways (Fig. 1C and D). Considering the crucial roles of lysosomes in innate immunity-based responses to intracellular pathogens, we analyzed the enriched genes and found a total of 13 lysosome-associated genes that were significantly (*FDR* ≤ 0.05) upregulated in the LEW rat (but not in BN rats) following *T. gondii* infection (Table 1). Furthermore, by gene ontology analysis, we found that, in response to *T. gondii* infection, the LEW rat enriches distinct molecular functions that are different from the BN rat’s (Fig. S1A through D). Consistent with our previous observation that, unlike the BN rat, the LEW rat upregulates GIMAPs in response to *T. gondii* infection (5), by gene ontology enrichment analysis, we found that GTPase activity was among the top 10 enriched molecular functions (Fig. S1B and C).

**TABLE 1:** Upregulated lysosome-associated genes in LEW compared with BN rats in response to *T. gondii* infection

Ensembl gene number	Gene	Fold change in expression (FDR)				Subcellular location^*b*^
		BN_Toxo_ vs. BN_PBS_^*a*^	LEW_Toxo_ Vs. LEW_PBS_^*a*^	LEW_Toxo_ vs. BN_Toxo_^*a*^	LEW_PBS_ vs. BN_PBS_^*a*^	
ENSRNOG00000008369	GIMAP 4	ND	4.6	2.6	ND	Cytosol/lysosomal process
ENSRNOG00000008416	GIMAP 5	ND	3.4	2.5	ND	Lysosomal membrane
ENSRNOG00000033338	GIMAP 6	ND	2.4	2.5	ND	Cytosol/lysosomal process
ENSRNOG00000009080	ATP6V1D	ND	1.7	1.4	ND	Function in lysosome
ENSRNOG00000017220	TCIRG1	ND	1.9	1.8	ND	Lysosome/cytoplasmic
ENSRNOG00000017871	Sidt2	ND	1.8	1.3	ND	Lysosomal membrane
ENSRNOG00000008258	Fnbp1	ND	2.0	ND	ND	Lysosome/cytoplasm
ENSRNOG00000002520	Litaf	ND	4.2	ND	ND	Lysosomal membrane
ENSRNOG00000006542	ATP6V0C	ND	2.0	ND	ND	Lysosomal vesicle
ENSRNOG00000014956	Slc11a1	ND	1.8	1.7	ND	Lysosomal membrane
ENSRNOG00000030199	Tmem192	ND	1.7	1.6	ND	Lysosomal membrane
ENSRNOG00000012247	Rab7a	ND	1.5	1.3	ND	Lysosomal membrane
ENSRNOG00000055439	Cln5	ND	1.5	1.3	ND	Lysosomal membrane
ENSRNOG00000012684	Bloc1s2	ND	1.4	ND	ND	Lysosomal membrane
ENSRNOG00000000962	Slc15a4	ND	1.5	ND	ND	Lysosomal membrane
ENSRNOG00000018433	Hps6	ND	1.5	ND	ND	Lysosomal membrane

^*a*^ BN_Toxo _and LEW_Toxo _(*T. gondii*-infected BN and LEW rats, respectively); BN_PBS_ and LEW_PBS _(uninfected BN and LEW rats, respectively). ND, not differentially expressed.

^*b*^ Subcellular location as reported in UniProt database (https://www.uniprot.org/uniprotkb/) for each Ensembl accession number.

### Knockout of ATP6V1D gene enhances intracellular *T. gondii* growth

**Fig 2:**
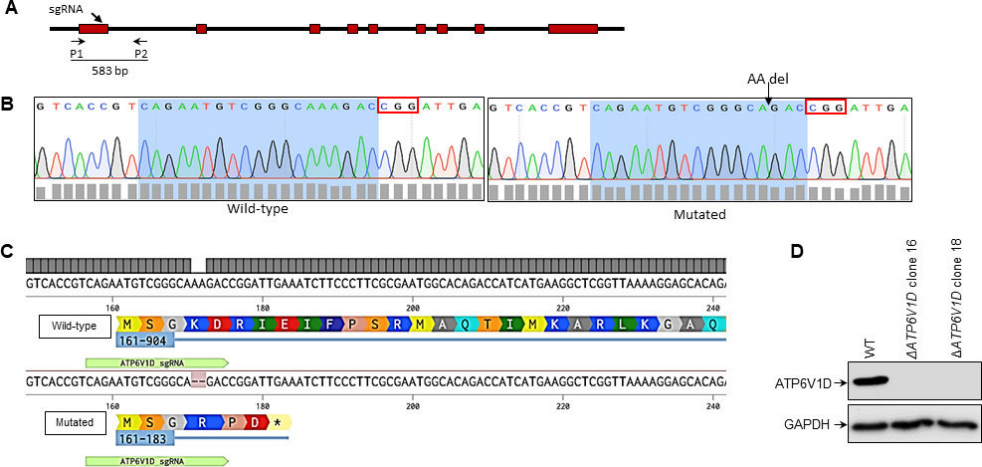
Generation of the ATP6V1D knockout (*∆ATP6V1D*) NR8383 cell line by CRISPR/Cas9 editing. (**A**) Schematic illustration of the ATP6V1D genomic locus structure in rat. The location of the CRISPR single guide RNA sequence (sgRNA) is shown by an arrow, indicating the predicted Cas9-induced DNA double-strand break within the first exon of the ATP6V1D gene verified by sequencing using primers P1 and P2 that encompass the sgRNA-targeting site. (**B**) Representative chromatograms of ATP6V1D genomic sequences from wild-type and mutant cells confirmed by sequencing. The full sgRNA sequence in the wild-type gene and the partial sequence in the mutated gene after biallelic homozygous deletion of two nucleotides are highlighted in blue. The red rectangle marks the PAM sequence. The probable site of Cas9 cleavage and the DNA repair pathway-induced two-nucleotide deletion are indicated by an arrow in the mutated sequence. (**C**) Sequence alignment of ATP6V1D mRNA from wild-type and genetically modified NR8383 macrophages showing the corresponding translated proteins. An early stop codon (*) in the reading frame of the mutated ATP6V1D mRNA results in the translation of a short-chain peptide instead of the complete protein. (**D**) Western blotting analysis of the expression of ATP6V1D protein in wild-type and homozygous knockout NR8383 macrophage cell lines using an anti-ATP6V1D antibody. GAPDH protein was used as a loading control. In the representative blot shown, the ATP6V1D protein is identified in the wild-type NR8383 cells but is absent in the ∆ATP6V1D NR8383 cells.

**Fig 3:**
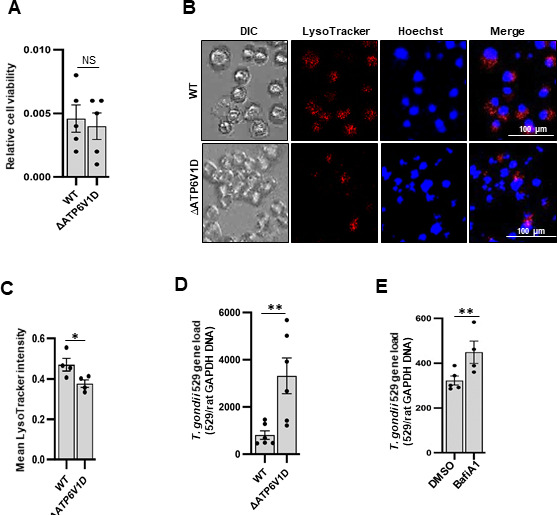
Analysis of the phenotypic features of the wild-type (WT) and the ATP6V1D knockout (*∆ATP6V1D*) NR8383 cells *in vitro*. (**A**) Comparison of the viability of the WT and *∆ATP6V1D* NR8383 cells after 48 h of culture using the cell proliferation reagent WST-1 assay. The relative cell viability was derived by subtracting the background absorbance in wells with growth medium only (without cells) from the test wells. (**B**) Fluorescence microscopic analysis of LysoTracker Red staining of the intracellular acidification of WT and *∆ATP6V1D* cells in culture. DIC: differential inference contrast; Hoechst: cell nucleus stain (blue); LysoTracker: acidification depicted by red; merge: overlay of panels. (**C**) Quantification of the intracellular acidification signal (red staining) in WT and *∆ATP6V1D NR8383* cells. For each biological replicate (represented by a single data point), LysoTracker intensity was quantified from 30 independent microscopic fields and averaged to obtain a single data point. (**D**) Quantitative real-time PCR analysis of the load of *T. gondii* in WT and *∆ATP6V1D* cells 48 h post-infection *in vitro*. (**E**) Quantification of *T. gondii* load in WT NR8383 cells with 5 nM final concentration of v-ATPase inhibitor Bafilomycin A1-treatment (BafiA1) or with 0.4% (v/v) DMSO for 24 h post-parasite invasion of host cells. Total genomic DNA was extracted from whole-cell cultures, and equal amounts of DNA were used as the template for qPCR analysis of the *T. gondii* 529 repetitive gene and normalized with the rat GAPDH gene load. Each data point is a biological replicate. Error bars represent SEM of the mean of the data points. Statistical significance differences between groups are depicted by asterisks (*, *P* < 0.05; **, *P* < 0.001; NS, not significant).

Our transcriptomic gene ontology enrichment analysis revealed that among the upregulated lysosome-associated genes in LEW rats in response to *T. gondii* infection was ATP6V1D that encodes a subunit of the lysosomal v-ATPase proton pump (Table 1). To determine the role of ATP6V1D, we generated a ∆*ATP6V1D* mutant rat macrophage cell line via CRISPR/Cas9 genome editing (Fig. 2A). The ATP6V1D gene locus-targeted mutation was confirmed by sequencing (Fig. 2B) and bioinformatic analysis of the mutant translated gene (Fig. 2C). Western blotting analysis validated the absence of the ATP6V1D protein in the mutant cell line (Fig. 2D). By using the cell proliferation reagent (WST1) to analyze the effect of ATP6V1D knockout on the viability of the cells, there was no notable difference between the wild-type and mutant NR8383 cells (Fig. 3A).

As ATP6V1D is a component of the v-ATPase proton pump that drives H+ from the cell cytosol into the lysosome lumen (13, 14), we analyzed the effect of ATP6V1D knockout on lysosomal acidification. By using the LysoTracker Deep Red staining assay, we found that LysoTracker staining intensity was significantly (*P* < 0.05) reduced in the ∆*ATP6V1D* compared with the wild-type NR8383 cells (Fig. 3B and C), indicating that knockout of ATP6V1D led to reduced acidification of the lysosomal lumen. As the next logical step, we determined the effect of impaired lysosomal acidification on intracellular *T. gondii* growth. We cultured *T. gondii*-infected wild-type and ∆*ATP6V1D* NR8383 cells for 48 h followed by quantification of parasite growth in the cultures by quantitative real-time PCR (qPCR). We observed over a threefold increase in the rate of parasite growth in the ∆*ATP6V1D* compared with the wild-type cells (Fig. 3D), despite the similar initial invasion rates of *T. gondii* in both cell lines (Fig. S2). As an alternative approach, we analyzed the effect of bafilomycin A1, a potent v-ATPase inhibitor, at a concentration not toxic to mammalian cells (16, 18), and found that wild-type NR8383 cells treated with 5 nM bafilomycin A1 had increased rate of intracellular *T. gondii* growth (Fig. 3E), consistent with impaired v-ATPase pump activity, leading to reduced control of intracellular *T. gondii* proliferation. Corroboratively, we observed that bafilomycin A1 treatment of the human v-ATPase C subunit (Fig. S3A) in foreskin fibroblast (HFF) cells also resulted in increased intracellular growth of *T. gondii* (Fig. S3B).

### GIMAP transgene overexpression functionally mitigates impaired lysosome acidification through increased lysosome recruitment

**Fig 4:**
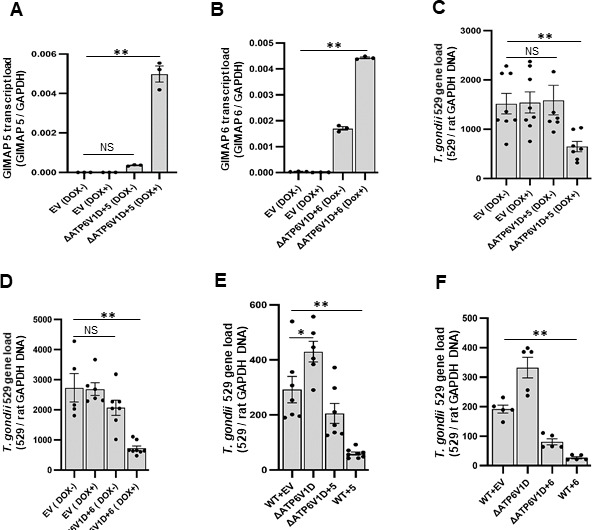
Effect of upregulated expression of GIMAP transgenes on growth of *T. gondii* in *ΔATP6V1D* and wild-type (WT) NR8383 cells. Real-time PCR quantification of (**A**) GIMAP 5 (ΔATP6V1D+5), and (**B**) GIMAP 6 (ΔATP6V1D+6) transcripts in *ΔATP6V1D* NR8383 cells engineered for doxycycline-inducible expression of transgene or empty pLVX-TetOne-Puro vector (EV). Cells were cultured for 24 h with or without doxycycline treatment (DOX+ or DOX-, respectively), followed by total RNA extraction and synthesis of cDNA that was used for transcript quantification and normalization with rat GAPDH. (**C–F**) Analysis of *T. gondii* growth in *ΔATP6V1D* and WT NR8383 cells with or without induced expression of GIMAP 5 or 6 transgenes. *ΔATP6V1D* NR8383 cells with GIMAP 5 or 6 transgene (∆ATP6V1D+5 or ∆ATP6V1D+6), and WT NR8383 cells with GIMAP 5, GIMAP 6, or empty expression vector (WT+5, WT+6, or WT+EV) were infected with *T. gondii* 24 h post-doxycycline induction and incubated for a further 48 h, followed by extraction of genomic DNA and real-time PCR quantification of the *T. gondii* 529 repetitive gene fragment that was normalized with the rat GAPDH gene fragment. Each data point is a biological replicate. Error bars represent SEM of the mean of the data points. Statistical significance differences between treatment groups are indicated with asterisks (*, *P* < 0.05; **, *P* < 0.001; NS, not significant).

We have previously reported that overexpression of GIMAP (5 or 6) transgenes in wild-type NR8383 cells promotes translocation of lysosomes to the PVM, with concomitant restriction of intracellular *T. gondii* proliferation (5). Therefore, in the present study to explore the interplay between GIMAPs and v-ATPase functions in modulating intracellular *T. gondii* growth, we analyzed the effects of GIMAP transgene overexpression on the growth of *T. gondii* in ∆*ATP6V1D* NR8383 cells. To achieve this, we first generated ∆*ATP6V1D* NR8383 cell lines for doxycycline-inducible overexpression of GIMAP 5 or 6 transgenes. By qPCR analysis of the transgene-engineered ∆*ATP6V1D* NR8383 cell, we found that following doxycycline induction, GIMAP 5 transcript levels were upregulated by 4.5-fold (Fig. 4A), while those of GIMAP 6 were upregulated by threefold (Fig. 4B) above baseline levels. Importantly, we have previously found that the concentration of doxycycline (1 μg/mL) used for transgene induction does not affect the intracellular growth of *T. gondii in vitro* (5). Therefore, we performed infection assays using the engineered ∆*ATP6V1D* NR8383 cell lines with or without doxycycline induction of GIMAP transgenes. We found that 48 h post-infection, ∆*ATP6V1D* cells overexpressing GIMAP 5 transgene had approximately 2.5-fold lower *T. gondii* load than those expressing baseline levels (Fig. 4C). Similarly, in GIMAP 6 transgene-overexpressing ∆*ATP6V1D* NR8383 cells, there was a fivefold lower *T. gondii* load than in the control cells (Fig. 4D), suggesting that GIMAPs overexpression functionally mitigated the impaired v-ATPase-associated increase in intracellular *T. gondii* growth. However, while overexpression of GIMAP 5 or 6 transgenes decreased parasite growth in both wild-type and ∆*ATP6V1D* NR8383 cells, the reduction was notably about threefold more in the wild-type than in the ∆*ATP6V1D* NR8383 cell (Fig. 4E and F), suggesting that GIMAPs and v-ATPase synergize in restricting intracellular *T. gondii* growth.

**Fig 5:**
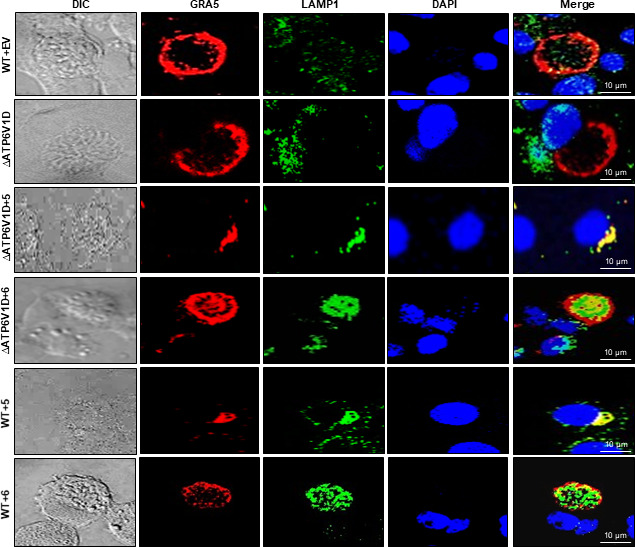
Immunofluorescence analysis of the localization of the parasitophorous vacuole membrane marker (GRA5) and lysosome marker (LAMP1) proteins in *T. gondii*-infected wild-type (WT) and ATP6V1D knockout (∆ATP6V1D) NR8383 cells with or without GIMAP transgene overexpression. *∆ATP6V1D* cells engineered for inducible overexpression of GIMAP 5 (∆ATP6V1D+5), GIMAP 6 (∆ATP6V1D+6), or WT cells with empty pLVX-TetOne-Puro expression vector (EV), and WT NR8383 cells with GIMAP 5 or GIMAP 6 Transgenes (WT+5 and WT+6, respectively) were induced with doxycycline for 24 h and, thereafter, infected with *T. gondii* and incubated for a further 48 h. Immunofluorescence assays were performed on the cells using a combination of LAMP1 antibody (green), GRA5 antibody (red), and DAPI nuclear stain (blue).

**Fig 6:**
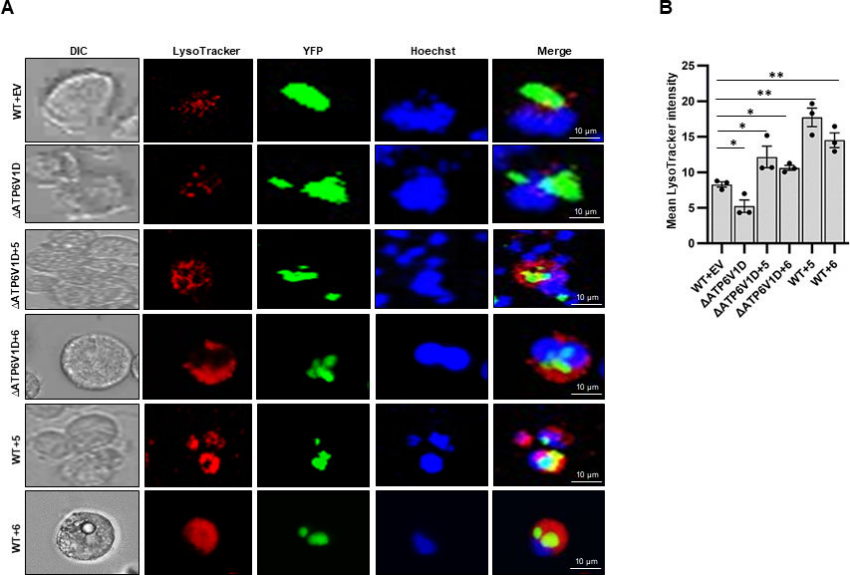
Fluorescence microscopic analysis of parasite vacuole acidification in *T. gondii*-infected wild-type (WT) and ATP6V1D knockout (∆ATP6V1D) NR8383 cells with or without GIMAP transgene overexpression. (**A**) *∆ATP6V1D* cells engineered for inducible overexpression of GIMAP 5 (∆ATP6V1D+5) or GIMAP 6 (∆ATP6V1D+6), and WT cells with empty pLVX-TetOne-Puro expression vector, GIMAP 5 or 6 (WT+EV, WT+5 and WT+6, respectively) were induced with doxycycline for 24 h, followed by infection with *T. gondii* and incubation for a further 48 h. The cultures were treated with Hoechst stain (blue) and LysoTracker Deep Red (red) and analyzed by fluorescence microscopy. The green color represents intracellular *T. gondii* tachyzoites constitutively expressing yellow fluorescent protein (YFP). (**B**) The intensity of the LysoTracker stain within parasite vacuoles of host cells randomly selected per microscopic field was measured by densitometry using ImageJ software. The Mean LysoTracker intensity is the average intensity quantification from 30 different microscopic fields for each data point. Error bars represent SEM of the mean of the data points. Levels of statistical significant difference in LysoTracker intensity are depicted by asterisks (*, *P* < 0.05; **, *P* < 0.001).

Furthermore, by immunofluorescence co-localization of LAMP1, a lysosomal marker protein (16), with GRA5, a PVM marker (19, 20), 48 h post-infection, we found that the wild-type and ∆*ATP6V1D* cells had granular and sparse cytoplasmic distribution of lysosomes that did not seem to overlap with prominent PVs (Fig. 5). On the contrary, ∆*ATP6V1D* and wild-type cells overexpressing GIMAP 5 or 6 transgene had notably smaller PVs that densely overlapped with lysosomes (Fig. 5), as corroborated by relative quantification of the overlap of the PVM marker protein (GRA5) and the lysosome marker, LAMP1 (Fig. S5). Because lysosomal enzymes are activated by the acidic intraluminal environment through the action of the v-ATPase proton pump (13, 14), we used the LysoTracker Deep Red dye assay to determine the acidification intensity of the overlap areas of lysosomes and PVs in infected cells. We found that acidification of the vacuoles was more prominent in both wild-type and ∆*ATP6V1D* cells overexpressing GIMAP 5 or 6 transgene than in the cells without transgene overexpression (Fig. 6A). However, comparatively, the intensity of intra-vacuole acidification in ∆*ATP6V1D* cells overexpressing GIMAP transgenes was lower than in the wild-type cells with GIMAP 5 or 6 transgene overexpression (Fig. 6B). Considering that there was still lysosomal acidification occurring in ∆*ATP6V1D* cells (though at a significantly reduced rate when compared with the wild-type cells), it can be inferred that impaired v-ATPase pump in ∆*ATP6V1D* cells was mitigated by upregulated GIMAP 5 or 6 that induced dense accumulation of lysosomes at the PVM (5), thus exposing intra-vacuole parasites to more lysosomal enzymes.

## DISCUSSION

Rats, like immunocompetent humans, develop a subclinical chronic infection but vary in their susceptibilities to *T. gondii* infection depending on the rat strain (21). The resemblance in the progression of toxoplasmosis between rats and humans warrants the use of rats as ideal quintessential models for dissecting host molecular mechanisms that are important for host resistance to *T. gondii* infection. Indeed, our analysis of the global transcriptomic data of the LEW rat (*T. gondii*-refractory rat strain) in comparison to its counterpart the *T. gondii*-permissive BN rat revealed that, in addition to having inherently upregulated immune-related pathways, the LEW rat upregulated lysosomal function-associated molecules and GTPase activity-related molecules including GIMAPs in response to *T. gondii* infection. GIMAPs possess small GTPase domains that are conserved among mammalian species (22–24). Interestingly, ectopic overexpression of GIMAPs in parasite-permissive rat or bovine macrophages induces translocation of lysosomes to the PVM with resultant inhibition of intracellular replication of *T. gondii* and *Neospora caninum,* respectively (5, 25). The fusion process of lysosomes to intracellular pathogen vacuole membranes is a crucial mechanism for facilitating lysosomal hydrolytic enzyme-based destruction of the intra-vacuole pathogens (12, 26, 27). Notably, the activation of the lysosomal hydrolytic enzymes is achieved by the translocation of H+ from the cell cytosol into the lysosome lumen through the function of the v-ATPase proton pump (13, 14).

Herein, we specifically investigated the interplay between the upregulation of expression of GIMAP 5 and 6 and the role of the v-ATPase proton pump in modulating intracellular parasite growth in a *T. gondii*-permissive rat macrophage cell line (NR8383). The v-ATPase consists of a peripheral V1 domain that hydrolyzes ATP, and a lysosomal membrane integral V0 domain that translocates protons into the lysosomal lumen, thereby lowering intraluminal pH and activating lysosomal hydrolytic enzymes (13, 14). ATP6V1D, as a component of the V1 domain, is required for lysosome acidification (15, 16, 28). Corroboratively, disruption of ATP6V1D has been shown to impair v-ATPase function, leading to defective lysosomal acidification, which in turn compromises overall lysosomal enzymes’ degradative capacity (14, 16, 29, 30). Consistently, we observed that ATP6V1D knockout in the rat macrophage NR8383 cell line resulted in impaired lysosomal acidification, with concomitant increase in intracellular *T. gondii* replication beyond the wild-type NR8383 cell line’s inherent permissiveness. This implied that, even in the *T. gondii*-permissive NR8383 rat macrophages, baseline expression of ATP6V1D does modulate the rate of parasite growth to some extent. Intriguingly, in the *T. gondii*-refractory LEW rat, we found upregulated expression of ATP6V1D (in response to infection) above baseline levels seen in the permissive BN rat, thus suggesting a correlation between ATP6V1D expression level and the extent of inhibition of intracellular parasite growth. The ATP6V1D, as a subunit of the ATPase proton pump, is part of the central rotor axle that propels the V0 subcomplex to shuttle protons across the lysosomal membrane into the lumen (13, 14), and is thus required for lysosomal acidification (15, 16). Corroboratively, we found that treatment of wild-type NR8383 and HFF cells with bafilomycin A1, a v-ATPase inhibitor, inhibited lysosomal acidification (16, 18, 29, 31). Bafilomycin A1 blocks the V0 domain’s subunit C, which is highly conserved in rat and human cells, and is driven by the central axel (which comprises ATP6V1D) to move protons across the lysosomal membrane into the lumen (13, 14). Therefore, disruption of either ATP6V1D or V0 domain would impair the v-ATPase proton pump function (16, 18, 32). Interestingly, the V1 and V0 domains of the v-ATPase proton pump can dynamically assemble or disassemble in response to various stimuli (33, 34), thus allowing the proton pump to function on-need basis (18).

While the phagosome-lysosome fusion process serves as an important mechanism for the destruction of invading intracellular microbes by the lysosomal hydrolytic enzymes that are discharged into the phagosome following fusion (12), the *T. gondii* PVM is non-fusogenic resulting in the protection of the intracellular parasites from destruction by lysosomal enzymes (35). However, we have previously shown that in the *T. gondii*-refractory LEW rat, upregulation of GIMAP 4, 5, or 6 in response to *T. gondii* infection induces translocation of lysosomes to the PVM (5). Notably, in the present study, we found that overexpression of GIMAP 5 or 6 rat transgenes in a *T. gondii*-permissive rat macrophage cell line (NR8383) induced dense localization of lysosomes on the PVM following *T. gondii* infection, leading to intense intra-vacuole acidification with concomitant restriction of parasite growth. Interestingly, the intensity of the upregulated GIMAP-induced intra-vacuole acidification was lower in the ∆*ATP6V1D* than in the wild-type NR8383 cell line. This implied that, while the upregulation of GIMAPs drives lysosomes to fuse with the otherwise non-fusogenic PVM, a fully functional v-ATPase is required for a robust activation of the lysosomal enzymes that would then degrade the intra-vacuole parasites. GIMAP 5 contains a C-terminal anchor that interacts with lysosomes (36, 37) and has been shown to regulate lysosomal Ca^2+^ accumulation in T lymphocytes (38). Furthermore, lysosomal Ca^2+^ has been demonstrated to modulate the release of lysosomal cytotoxic granules (39), thus implicating GIMAP 5 in playing a role in the release of lysosomal hydrolytic enzymes. Consistently, GIMAP 5, ATP6V1D, and LAMP1 proteins have been shown to colocalize within the lysosomal membrane (16, 36, 38, 40). In addition, a lysosomal membrane-associated protein complex called Ragulator has been reported to interact with small GTPase domain-containing family of proteins (41), thus corroborating our findings that GIMAP 5 and 6 (that are small GTPase proteins) colocalize with the lysosome marker protein, LAMP1.

In conclusion, it can be inferred that while upregulated GIMAPs activate lysosome translocation to the PVM, the upregulation of the function of the v-ATPase pump plays an additively significant role in parasite growth restriction through enhanced acidification of the intraluminal compartment of lysosomes that in turn activates lysosomal hydrolytic enzymes to degrade intra-vacuole parasites. Thus, our findings that upregulated expression of GIMAPs drives lysosomal-PVM fusion, and that upregulated ATP6V1D facilitates lysosomal intraluminal acidification, indicate synergy between GIMAPs and ATP6V1D in restricting intracellular *T. gondii* growth.

## MATERIALS AND METHODS

### Parasite culture

The wild-type type I RH strain of *T. gondii* and the RH strain constitutively expressing cytosolic yellow fluorescent protein (42) used in this work were maintained in confluent human foreskin fibroblasts (HFF) cells that were cultured in Iscove’s modified Dulbecco’s medium supplemented with 10% (vol/vol) heat-inactivated fetal bovine serum, 1% (vol/vol) GlutaMAX, and 1% (vol/vol) penicillin-streptomycin-amphotericin B (Fungizone) (Life Technologies, USA) at 37°C with 5% CO₂. *T. gondii* tachyzoites were extracted from infected HFF cells by detaching the cells and passing the cell suspension twice through a 25-gauge needle, followed by filtration through a 3-μm pore-size filter to isolate the tachyzoites from the cell debris. Isolated tachyzoites were washed three times in phosphate-buffered saline (PBS), and their concentration was determined using a hemocytometer.

### Transcriptomic gene ontology and pathway enrichment analysis

We have previously reported RNA sequencing data (GEO repository accession number GSE100203) for pairwise comparisons of global gene expression in LEW and BN rats with or without *T. gondii* infection (5, 17). In the present study, analysis of the RNA sequencing data in the GEO repository was performed across four different treatments: (i) infected BN vs uninfected BN rats (BN_Toxo_ vs BN_PBS_), (ii) infected LEW vs uninfected LEW rats (LEW_Toxo_ vs LEW_PBS_), (iii) infected LEW vs infected BN rats (LEW_Toxo_ vs BN_Toxo_), and (iv) uninfected LEW vs uninfected BN rats (LEW_PBS_ vs BN_PBS_). Differentially expressed genes (DEGs) between the groups were defined as those with adjusted *P*-values of ≤0.05 and |log₂ fold change| ≥ 1.2. The Database for Annotation, Visualization, and Integrated Discovery (DAVID) bioinformatics tool (v6.8) was used to determine the top ten gene ontology immune-related pathways and enriched molecular functions. DEGs with adjusted *P*-values (*P* ≤ 0.05) from each pairwise comparison were uploaded to DAVID using their official Ensembl Gene identification numbers. The top 10 immune-related pathways were determined using immune-associated gene ontology terms, with a cutoff of −log_2_ ≥ 1.5. Similarly, the top 10 enriched molecular function pathways were selected based on their lowest calculated *P*-value from the DAVID gene ontology database. Statistical significance of pathway enrichment was determined using Fisher’s exact test with Bonferroni correction for multiple comparisons. An individual pathway containing at least 15 genes with *P* ≤ 0.05 was considered.

### Generation of sgRNAs and CRISPR plasmid for rat ATP6V1D knockout

Deletion of the ATP6V1D gene in the NR8383 rat macrophage cell line (CRL2192; ATCC) was performed using CRISPR/Cas9 genome editing with the GeneArt CRISPR Nuclease Vector and CD4 Enrichment kits, following the manufacturer’s instructions (Invitrogen, Waltham, MA). Briefly, a target-specific single guide RNA (sgRNA) with fewer off-target sites was designed using the Benchling tool (www.benchling.com) to target the rat ATP6V1D gene (NCBI Gene number 299159). Two single-stranded DNA oligonucleotides (Table 2) for amplification of CRISPR sgRNA with suitable 3′ overhangs for directional cloning into the linearized GeneArt CRISPR Nuclease Vector (with CD4 reporter) were synthesized by Integrated DNA Technologies (USA). The generated recombinant GeneArt CRISPR vector bearing the sgRNA for ATP6V1D was transformed into One Shot chemically competent TOP10 *E. coli* cells (Invitrogen, Waltham, MA) for overnight culture. Ampicillin-resistant colonies were selected for plasmid DNA extraction using the Wizard Plus SV Minipreps DNA Purification Kit (Promega, Madison, WI). Each positive transformant’s CRISPR nuclease construct was then checked by Sanger sequencing for the correct sgRNA sequence using the U6 Forward primer. After confirming successful cloning, the NucleoBond Xtra Midi kit (MACHEREY-NAGEL, Allentown, PA) was used to extract high-quality CRISPR plasmid DNA for transfection.

**TABLE 2:** Oligonucleotides used in this study

Primer name	Sequence (5′ to 3′)
ATP6V1D sgRNA-F	CAGAATGTCGGGCAAAGACGTTTT
ATP6V1D sgRNA-R	GTCTTTGCCCGACATTCTGCGGTG
U6 forward	GGACTATCATATGCTTACCG
P1	CTGTCCTCTGGCAACAGGAAG
P2	GTCTCATCCCTCAGCACTGATG
T7 pro	TAATACGACTCACTATAGGG
Sp6 pro	ATTTAGGTGACACTATAG
GIMAP 5_201-F	CCCTCGTAAAGAATTCATGGAGGACCATGGCTT
GIMAP 5_201-R	GAGGTGGTCTGGATCCTCATTTCCACCTGCCAAT
GIMAP 6-F	CCCTCGTAAAGAATTCATGAATTGGCTTTACAGTAAAAC
GIMAP 6-R	GAGGTGGTCTGGATCCTTAAAGGGTTTTGCTGGAGA
pLVX-F	ATGTAAACCAGGGCGCCTAT
pLVX-R	ACCCGTCTTTGGATTAGGCA
529-F	AGCTGCGTCTGTCGGGATGAGA
529-R	ACCCTCGCCTTCATCTACAGT
Rat-GAPDH-F	TTCCCTGAGTCCTATCCTGGGAA
Rat-GAPDH-R	TTATAGGAACTGGATGGTGGGGG
RT-GIMAP5-F	GCTTCCTAGTGGTGGACACG
RT-GIMAP5-R	AGTTGGGTCACCAGCAACAA
RT-GIMAP6-F	CAAAATCAGCGCTCGACCAG
RT-GIMAP6-R	GGGGTGTCGATCACCTCAAG
Rat-GAPDH-mRNA-F	GGATACTGAGAGCAAGAGAGA
Rat-GAPDH-mRNA-R	GGGTGCAGCGAACTTTAT

### Nucleofection and enrichment of transfected cells

About 2 × 10^6^ NR8383 cells cultured in IMDM medium supplemented with MEM 1× non-essential amino acid solution (Sigma), 1× OPI Media supplement-Hybri-Max (Sigma), 1.5 mg/mL sodium bicarbonate, 10% (v/v) heat-inactivated fetal bovine serum (Gibco, certified tetracycline-free), 100 U/mL penicillin, and 100 µg/mL streptomycin were harvested and electroporated with 5 µg of the purified recombinant CRISPR plasmid using the SF Cell Line 4D-Nucleofector X Kit and the EP-100 program on the Amaxa 4D-Nucleofector System (Lonza, Morristown, NJ) according to the manufacturer’s instructions. After nucleofection, the cells were immediately resuspended in modified (with 15% FBS) supplemented IMDM medium and transferred to a six-well plate for incubation in a humidified 37°C/5% CO_2_ incubator, undisturbed for 48 h, until transfected cells were enriched. Cell populations successfully transfected with the recombinant GeneArt CRISPR Nuclease Vector (with CD4 reporter) were enriched using the Dynabeads CD4 Positive Isolation Kit (Invitrogen, Waltham, MA), following the manufacturer’s instructions. One-half of the CD4+ enriched cells were reseeded and cultured at 37°C for 2 weeks, while the other half was used to assess CRISPR/Cas9-mediated double-stranded cleavage efficiency using the GeneArt Genomic Cleavage Detection Kit (Invitrogen, Waltham, MA) and primers P1 and P2 (Table 2) flanking the genomic region encompassing the CRISPR target site of the ATP6V1D gene locus.

### Clonal expansion and sequence confirmation of knockout cell lines

After expanding reseeded CD4+ enriched cells, single cells were cloned by limiting dilution. Following at least 4 weeks of clonal growth in filtered conditioned medium, cells from each clone were harvested to extract genomic DNA with the QuickExtract DNA Extraction Solution (Lucigen, Middleton, WI), according to the manufacturer’s instructions. PCR was then performed on the extracted DNA to amplify the target locus using P1 and P2 primers (Table 2), followed by cloning of the allelic PCR products into the pGEM-T Easy Vector (Promega, Madison, WI) and transformation into NEB Turbo Competent *E. coli* (New England Biolabs, Ipswich, MA). Random colonies were selected for plasmid isolation and subsequent DNA sequencing using the T7 Pro and SP6 Pro primers.

### Protein extraction and western blotting

To assess ATP6V1D expression, total protein was extracted from 10^6^ harvested NR8383 cells using ice-cold M-PER Mammalian Protein Extraction Reagent (Thermo Fisher Scientific, Waltham, MA) containing 1× EDTA free protease inhibitor cocktail (Thermo Fisher Scientific, Waltham, MA). Cell lysates were centrifuged at 16,000×*g* for 15 min at 4°C to remove cell debris, and then the supernatants were collected for calculating protein concentration using a Qubit 3.0 fluorometer (Life Technologies). M-PER reagent was used to normalize total protein concentration across samples. Equal amounts of protein samples were separated by SDS-PAGE and transferred to PVDF membranes. A mouse anti-v-ATPase D monoclonal antibody (sc-390384; Santa Cruz Biotechnology, Dallas, TX) was used at a 1:500 dilution to detect ATP6V1D. The secondary antibody used was an HRP-conjugated goat anti-mouse IgG (31,430; Invitrogen, Waltham, MA) used at a 1:5,000 dilution. Additionally, goat anti-GAPDH polyclonal antibody (PA1-9046; Invitrogen, Waltham, MA) was used at a 1:1,000 dilution as a loading control after stripping the membrane with Restore Western Blot Stripping Buffer (Thermo Fisher Scientific, Waltham, MA), and an HRP-conjugated donkey anti-goat IgG (A16005; Invitrogen, Waltham, MA) was used as the secondary antibody at a 1:5,000 dilution. Peroxidase activity was detected using the Clarity Western ECL substrate (Bio-Rad, Hercules, CA), and images were captured using a FluoroChem R imager (Protein Simple).

### Cell viability assays

Wild-type (WT) and Δ*ATP6V1D* NR8383 cells were seeded in 96-well plates and incubated for 48 h at 37°C with 5% CO_2_, following which 10 μL of the Cell Proliferation reagent, WST-1, was added to each well and incubated for a further 1 h, and 150 μL of the culture medium from each well transferred to a new 96-well plate. The absorbance of the formazan dye produced by metabolically active cells was measured at a wavelength of 420 nm using a scanning multi-well spectrophotometer (Spectra Max 384 Plus, Molecular Devices, USA). The background absorbance was obtained from growth medium incubated in cell-free wells. The cell viability was calculated as the relative absorbance value from test wells after subtracting the background absorbance in wells with growth medium but without cells.

### *T. gondii* invasion assay

To assess the rate of invasion of host cells by *T. gondii* tachyzoites, equal numbers of WT and Δ*ATP6V1D* mutant NR8383 cells were cultured to confluence in supplemented IMDM medium in eight-well cover Permanox slides (Thermo Fisher Scientific). Confluent monolayers were infected with freshly extracted *T. gondii* type I RH strain tachyzoites constitutively expressing Yellow Fluorescent Protein (YFP) at an MOI of 1:20 and incubated for 1 h at 37°C. Cells were then fixed with 3% (w/v) formaldehyde in PBS for 30 min at room temperature. Following washing with blocking buffer (3% bovine serum albumin in PBS), cells were incubated overnight at 4°C in blocking buffer containing mouse anti-SAG1 (C65620M; Meridian Life Science) at 1:500 dilution. The cells were later incubated for 1 h at room temperature with Alexa Fluor 594-conjugated goat anti-mouse immunoglobulin antibody (A11032; Invitrogen) as secondary antibodies diluted in blocking buffer at 1:500. The cells were air-dried, a drop of ProLong Gold antifade reagent with DAPI (4′,6-diamidino-2-phenylindole; Life Technologies) was added, and a coverslip was placed and sealed with nail polish. The cells were analyzed by fluorescence microscopy to determine the number of green+/red- (intracellular tachyzoites) and green+/red+ (extracellular tachyzoites) in 15 microscopic fields for each category. The percent invasion was derived as the average number of green+/red- tachyzoites divided by the average total tachyzoites (green+/red- plus green+/red+) and multiplied by 100.

### Analysis of effect of v-ATPase inhibitor (bafilomycin A1) on *T. gondii* growth

Wild-type NR8383 and HFF cells were grown to confluence in supplemented IMDM in 24-well plates. At approximately 70% confluence, cells were infected with freshly extracted *T. gondii* type I RH strain tachyzoites at an MOI of 1:10. The infected cultures were incubated for 2 h to allow parasite invasion, after which the medium was replaced with fresh medium containing either 5 nM bafilomycin A1 or 0.4% (v/v) DMSO (control), and cells incubated for a further 12 h. Subsequently, the medium was replaced with fresh medium containing 2.5 nM bafilomycin A1 or 0.2% DMSO and cultured for another 12 h, after which cells were harvested, and genomic DNA extracted. The DNA concentration from each well was measured and made uniform across samples by dilution with sterile molecular-grade water. *T. gondii* load was then quantified by qPCR.

### Generation of Δ*ATP6V1D* NR8383 cells for inducible expression of rat GIMAP transgenes

The construction of the recombinant vectors for inducible expression of the full coding sequences of LEW rat GIMAP 5 and GIMAP 6 (Ensembl gene numbers: ENSRNOG00000008416 and ENSRNOG00000033338, respectively) in the pLVX-TetOne-Puro expression vector (Clontech) and the production of lentiviral particles carrying the GIMAP 5 and 6 expression constructs or empty pLVX-TetOne-Puro vector were done as we previously reported (5). To generate Δ*ATP6V1D* NR8383 cells for inducible overexpression of GIMAP 5, GIMAP 6, or the empty pLVX-TetOne-Puro empty vector, Δ*ATP6V1D* NR8383 cells were seeded in 10 cm diameter petri dishes and cultured in supplemented IMDM until they were about 70% confluent. The cells were treated with 4 µg/mL of polybrene to enhance lentiviral particle entry. About 200 μL of lentiviral suspensions (~10⁵ IFU) was added dropwise, followed by gentle mixing. After overnight culture, the medium was replaced with fresh medium containing 12 µg/mL puromycin and maintained for 3 weeks with replenishment of medium every 3 days. After 21 days of puromycin selection, resistant cells were cloned by limiting dilution. To confirm transgene expression in the clonal lines, the cells were cultured in supplemented IMDM medium with or without 1 µg/mL doxycycline for 24 h to induce transgene expression and then analyzed for mRNA transcripts by qPCR. Briefly, total RNA was extracted from the whole cell culture and 1 μg treated with DNase I kit (Invitrogen) and used to synthesize cDNA with the Superscript Reverse Transcription III kit (Invitrogen). The cDNA was used for quantification of rat GIMAPs and rat GAPDH transcripts with primer pairs RT-GIMAP5-F and RT-GIMAP5-R (for GIMAP 5), RT-GIMAP6-F and RT-GIMAP6-R (for GIMAP 6), or Rat-GAPDH-mRNA-F and Rat-GAPDH-mRNA-R (for rat GAPDH) (Table 2). Each qPCR reaction mixture contained 1 μL of cDNA template, 1 μL of primer mix (500 nM each primer), and 10 μL of PowerUp SYBR Green Master Mix (Applied Biosystems by Thermo Fisher Scientific), with the final volume made up to 20 μL with sterile nuclease-free water. Cycling consisted of an initial denaturation at 95°C for 10 min, followed by 45 cycles at 98°C for 15 s and at 60°C for 1 min, and a final melting curve analysis step. A similar assay was performed using 10-fold serially diluted GIMAP 5, 6, or GAPDH amplicons as templates to generate respective quantification standard curves. Reactions were run on QuantStudio 3 Real-Time PCR System (Applied Biosystems). Targeted GIMAP transcript concentrations were normalized using GAPDH transcripts.

### Analysis of the effect of GIMAP transgene expression on *T. gondii* growth in Δ*ATP6V1D* NR8383 cells

Transgenic WT and Δ*ATP6V1D* NR8383 cells engineered for inducible expression of GIMAP 5 or 6 transgenes, or the empty pLVX-TetOne-Puro expression vector was grown to confluence in supplemented IMDM medium in 24-well plates. The cells were treated with 1 μg/mL of doxycycline for 24 h, after which fresh medium containing 1 μg/mL doxycycline was added, and the cells inoculated with freshly extracted *T. gondii* type I RH strain tachyzoites at an MOI of 1:20. At 48 h post-infection, genomic DNA was extracted from each well, and the concentration was measured and made uniform across samples by dilution with sterile molecular-grade water. Quantification of the amount of the *T. gondii* 529 repetitive gene fragment (GenBank accession number AF146527) and the rat GAPDH gene fragment (GenBank accession number BC029618) was performed by qPCR on equal amounts of DNA template. The primer pairs used were 529-F and 529-R for the 529 repetitive gene, and Rat-GAPDH-F and Rat-GAPDH-R for the GAPDH gene (Table 2). The primer pairs generated DNA fragments of 174 and 300 bp for the 529 repetitive and GAPDH genes, respectively. PCR products for each gene were fractionated on agarose gel and the DNA bands extracted using a QIAquick gel extraction kit (Qiagen). The concentration of the purified DNA fragments was measured, and 10-fold serial dilutions were generated and used as quantification standards for qPCR. Each qPCR reaction mixture contained 1 μL of DNA template, 1 μL of primer mix (500 nM of each primer), and 10 μL of PowerUp SYBR Green Master Mix, with the final volume made up to 20 μL with sterile nuclease-free water. Cycling conditions consisted of an initial denaturation at 95°C for 10 min, followed by 45 cycles of 98°C for 15 s and 60°C for 1 min, and concluded with a melting curve analysis. DNA quantities were derived by the system software using the generated standard curves. The relative amount of *T. gondii* was derived by dividing the concentration of the *T. gondii* 529 repetitive gene by the concentration of the rat GAPDH gene from each DNA sample.

### Immunofluorescence assays

Transgenic WT and Δ*ATP6V1D* NR8383 cells engineered for inducible expression of GIMAP 5 and 6 transgenes or the empty pLVX-TetOne-Puro expression vector were grown to confluence in supplemented IMDM medium in eight-well cover Permanox slides (Thermo Fisher Scientific). The cells were treated with 1 μg/mL of doxycycline for 24 h prior to infection, followed by replenishment with fresh medium containing 1 μg/mL doxycycline and inoculation with freshly extracted *T. gondii* type I RH strain tachyzoites at an MOI of 1:20. After 48 h of culture post-infection, cells were fixed with 3% (w/v) formaldehyde in PBS for 30 min at room temperature, followed by three washes with PBS, and permeabilization with 0.2% Triton X-100 in PBS at room temperature for 10 min. The cells were then treated with blocking buffer (0.1% Triton X-100 and 3% bovine serum albumin in PBS) for 1 h, followed by overnight incubation at 4°C in blocking buffer containing rabbit anti-LAMP1 antibody (ab24170; Abcam) and mouse monoclonal antibody against *T. gondii* GRA5 (BIO.018.6; Biotem) at 1:500 dilution. The cells were later incubated for 1 h at room temperature with Alexa Fluor 488-conjugated goat anti-rabbit antibody (A32731; Invitrogen) and Alexa Fluor 594-conjugated goat anti-mouse immunoglobulin antibody (A32742; Invitrogen) as secondary antibodies diluted in blocking buffer at 1:500, followed by three washes in PBS. The cells were air-dried, a drop of ProLong Gold antifade reagent with DAPI (4′,6-diamidino-2-phenylindole; Life Technologies) was added and a coverslip placed and sealed with nail polish. The slides were analyzed using a Nikon A1R confocal laser microscope system. The Pearson correlation coefficient between GRA5 and LAMP1 fluorescence intensity was calculated from at least 20 *T. gondii*-infected cells.

### LysoTracker deep red intracellular acidification assay

Transgenic WT and Δ*ATP6V1D* NR8383 cells engineered for inducible expression of GIMAP 5 and 6 transgenes or the empty pLVX-TetOne-Puro expression vector were grown to confluence in supplemented IMDM medium in eight-well cover Permanox slides. The cells were cultured in medium with 1 μg/mL of doxycycline for 24 h, following which the medium was replenished and the cultures inoculated with freshly extracted *T. gondii* type I RH strain tachyzoites constitutively expressing Yellow Fluorescent Protein (YFP) at an MOI of 1:20. At 48 h post-infection, the cultures were replenished with fresh medium containing 50 nM of LysoTracker Deep Red (Invitrogen) and 2 µg/mL of Hoechst 33,258 (Invitrogen). Following 30 min of incubation, the cultures were analyzed using a Nikon A1R confocal laser microscope system. The intensity of the LysoTracker Red for each test sample was measured from at least 30 different microscopic fields using ImageJ software and averaged to obtain a single data point.

### Statistical analyses

One-way ANOVA was used to compare the data across and between groups, while the false discovery rate method was used to perform multiple-hypothesis-test correction. A weighted gene correlation network analysis (WGCNA; v1.51) using the trimmed mean for M values (TMM) normalized log_2_ count per million (CPM) values was performed to compare gene expression patterns across treatment groups. All the sequencing data were analyzed using log_2_ scale values and then converted from the log_2_ fold change (FC) to the regular FC. All other experimental data analyses were performed using unpaired two-tailed Student’s *t* test with GraphPad PRISM v8 software, and *P*-values of 0.05 or less were considered significant.

## SUPPLEMENTAL MATERIAL

Fig. S1 spectrum.03947-25-s0001.tif Gene Ontology enrichment for molecular function.

Fig. S2 spectrum.03947-25-s0002.tif Analysis of the invasion rate of *T. gondii*.

Fig. S3 spectrum.03947-25-s0003.tif Effect of v-ATPase inhibitor bafilomycin A1 on *T. gondii* growth.

Fig. S4 spectrum.03947-25-s0004.tif Verification of inducible overexpression of GIMAPs.

Fig. S5 spectrum.03947-25-s0005.tif Pearson correlation coefficient analysis.

ASM does not own the copyrights to Supplemental Material that may be linked to, or accessed through, an article. The authors have granted ASM a non-exclusive, world-wide license to publish the Supplemental Material files. Please contact the corresponding author directly for reuse.
