# Supplementary figures and images for: Synergy between upregulated small GTPase immunity-associated proteins and lysosomal ATP6V1D in restricting intracellular Toxoplasma growth
Source: Microbiol Spectr. 2026 Apr 13;14(5):e03947-25. doi: 10.1128/spectrum.03947-25 (PMC13141973; doi:10.1128/spectrum.03947-25)

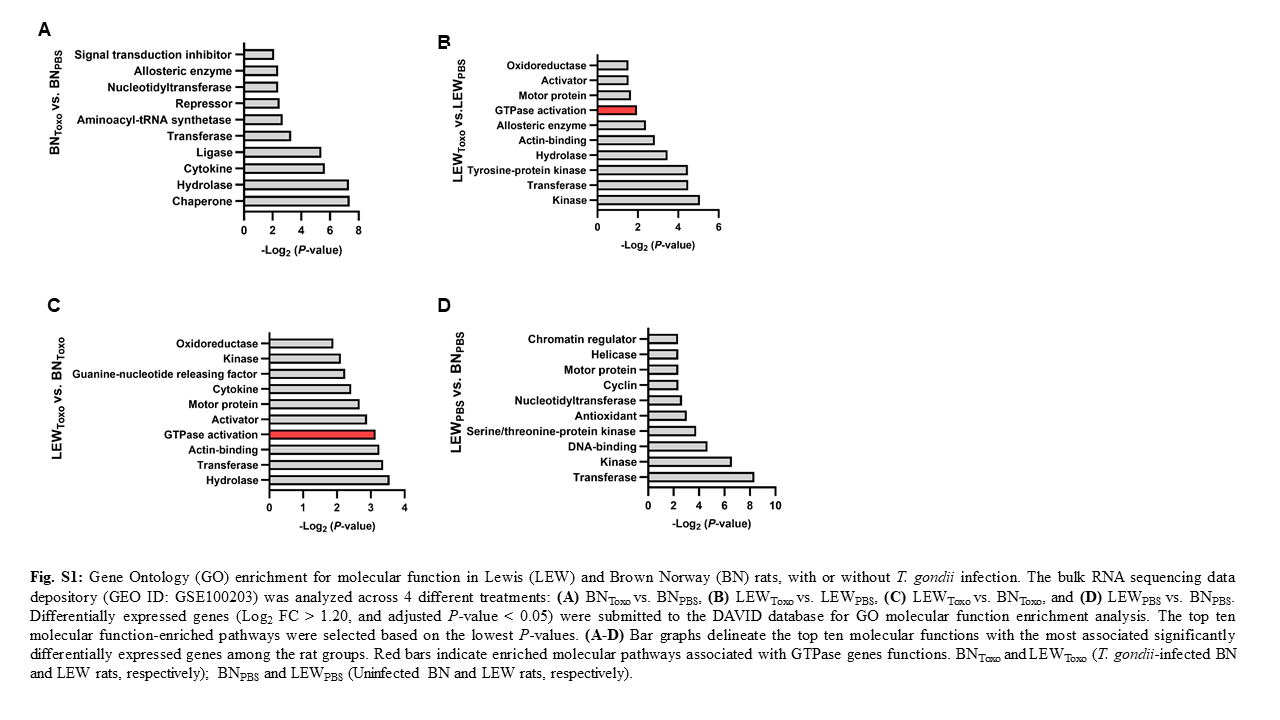

Supplement: Fig. S1 — Gene Ontology enrichment for molecular function. [file spectrum.03947-25-s0001.tif]

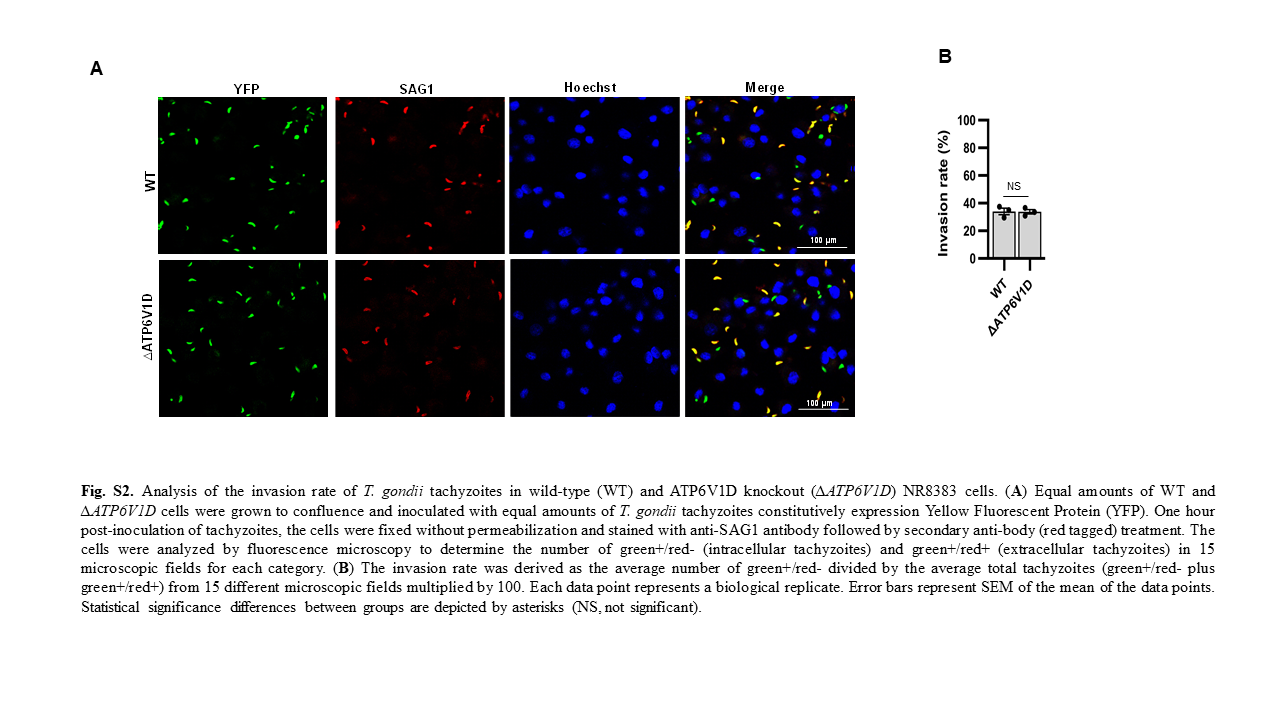

Supplement: Fig. S2 — Analysis of the invasion rate of T. gondii. [file spectrum.03947-25-s0002.tif]

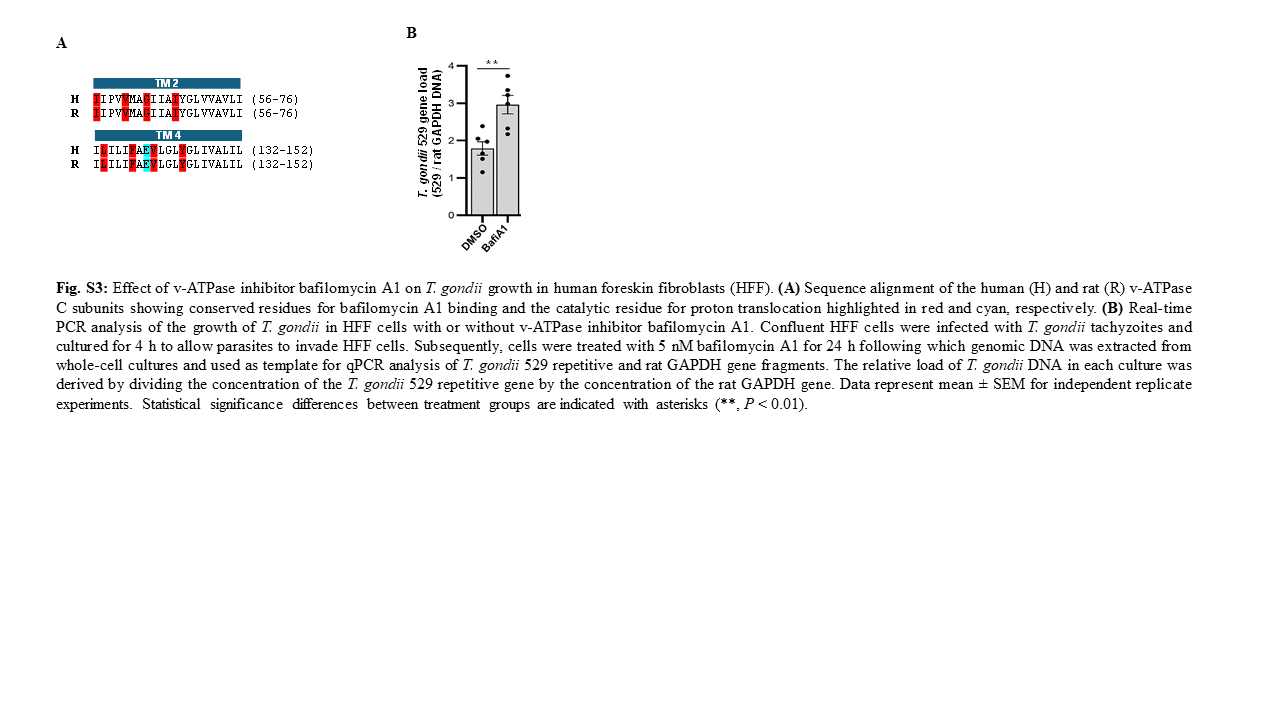

Supplement: Fig. S3 — Effect of v-ATPase inhibitor bafilomycin A1 on T. gondii growth. [file spectrum.03947-25-s0003.tif]

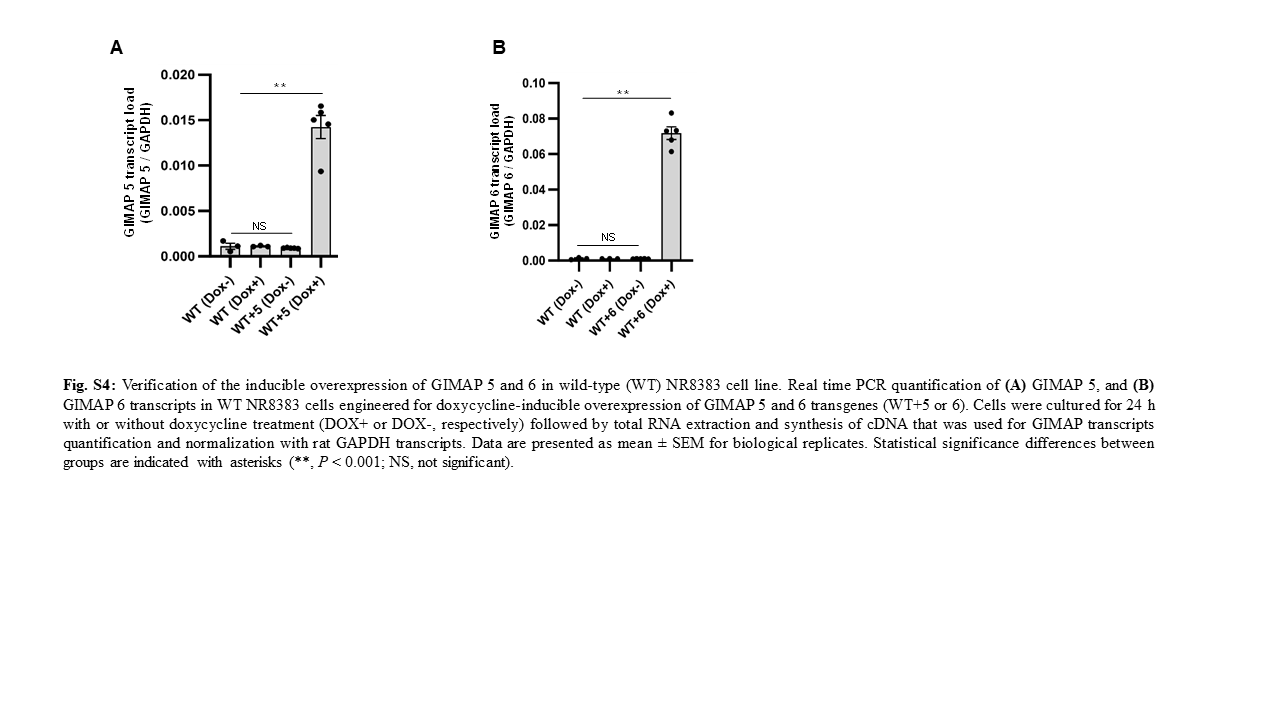

Supplement: Fig. S4 — Verification of inducible overexpression of GIMAPs. [file spectrum.03947-25-s0004.tif]

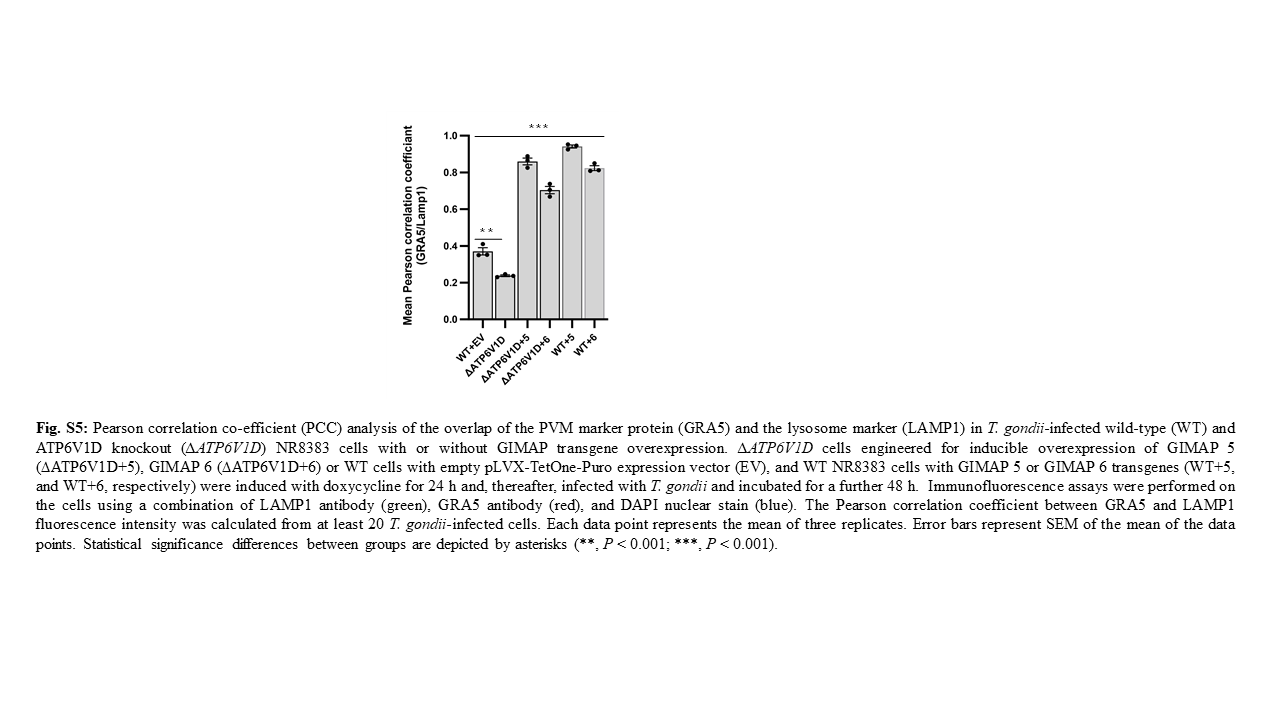

Supplement: Fig. S5 — Pearson correlation coefficient analysis. [file spectrum.03947-25-s0005.tif]
